# Inferring gene regulatory networks by thermodynamic modeling

**DOI:** 10.1186/1471-2164-9-S2-S19

**Published:** 2008-09-16

**Authors:** Chieh-Chun Chen, Sheng Zhong

**Affiliations:** 1 Department of Bioengineering, University of Illinois at Urbana Champaign, Urbana, IL 61801, USA; 2 Department of Computer Science, University of Illinois at Urbana Champaign, Urbana, IL 61801, USA; 3 Department of Statistics, University of Illinois at Urbana Champaign, Champaign, IL 61820, USA

## Abstract

**Background:**

To date, the reconstruction of gene regulatory networks from gene expression data has primarily relied on the correlation between the expression of transcription regulators and that of target genes.

**Results:**

We developed a network reconstruction method based on quantities that are closely related to the biophysical properties of TF-TF interaction, TF-DNA binding and transcriptional activation and repression. The Network-Identifier method utilized a thermodynamic model for gene regulation to infer regulatory relationships from multiple time course gene expression datasets. Applied to five datasets of differentiating embryonic stem cells, Network-Identifier identified a gene regulatory network among 87 transcription regulator genes. This network suggests that Oct4, Sox2 and Klf4 indirectly repress lineage specific differentiation genes by activating transcriptional repressors of Ctbp2, Rest and Mtf2.

## Background

Transcriptional control is a key regulatory mechanism for cells to direct their destinies. A large number of transcription factors (TFs) could simultaneously bind to a regulatory sequence. With the constellation of TFs bound, the expression level of a target gene is usually determined by the combinatorial control of a number of TFs. The interactions among regulatory proteins and their regulatory sequences collectively form a regulatory network. A major challenge in the study of gene regulation is to identify the interaction relationships within a regulatory network.

A number of analytical methods have been proposed to reconstruct gene regulatory networks from gene expression and protein-DNA binding data. Association rule mining [1], Boolean Network [2], temporal models [3,4], ARACNE [5] and Bayesian networks [6-8] are among the most popular routes. For example, the Module Networks approach built a probabilistic model for the gene expression correlations between regulators and target genes and iteratively searched for the most compatible partition of targets genes to their respective regulators [9]. The correlation of gene expression patterns of regulators and the target genes is often the essential piece of information utilized by the current procedures. It is widely recognized that the statistical correlation of the regulators and the targets is often an inaccurate representation of the regulator-target relationship [10,11]. This is because the quantity of a TF's mRNA does not necessarily correlate to its active protein concentration, and even the active protein concentration does not necessarily correlate to its transcriptional efficiency on every target gene. Using correlation, or some transformed version of correlation measure as the basis for reconstructing regulatory networks is an approximation made for convenience of modeling and analysis, with a sacrifice of making spurious findings (see examples in [9]). A network reconstruction method based on quantities that closely represent the biophysical properties of TF-DNA binding, transcription activation and repression is still missing.

Thermodynamic models of TF-DNA and TF-RNA polymerase (RNAP) interactions were pioneered by Buchler *et al*. [12] in prokaryotes. These models brought the stochastic interactions of TFs, regulatory sequences and RNAP into a statistical mechanics framework, and enabled a quantitative model for the transcription rate. Recently, Segal *et al*. [13] and our team [14] attempted to employ thermodynamic models in the study of eukaryotic gene regulation. Under a fix time point in drosophila development, Segal *et al*. demonstrated a thermodynamic model could predict the spatial expression patterns of segmentation genes in *Drosophila *[11]. In differentiating embryonic stem cells (ESCs), we showed that the interaction types of the TFs could be predicted from the temporal response of the target gene [14]. These successes made it tempting to experiment novel methods for reconstructing regulatory networks based on more biophysically appropriate metrics than correlation.

We describe here a computational framework, called Network-Identifier, for inferring regulatory networks from time course gene expression data. The gene expression values at each time point are supposed to be at an equilibrium state, which is a general setting for most of time course data available. Applying to the analysis of five datasets of differentiation of murine ESCs, we identified a transcription network composed of 34 TF-TF interactions and 185 TF-target relationships. Data from RNAi [15] and chromatin immunoprecipitation coupled with microarray (ChIP-chip) data [16,17] independently validated a statistically highly significant fraction of these regulatory relationships.

## Results

### Gene regulatory network in mouse ESCs

ESCs are derived from early mammalian embryos. ESCs possess two important characteristics that distinguish their importance in scientific and medical fields. First, they are capable of self-renewal through apparently unlimited, undifferentiated proliferation in cultured cell lines [18-20]; second, they have remarkable pluripotency potentials [21] to give rise to many different cell types in the body, which may contribute to the study of body development and regenerative medicine.

We employed five time series microarray datasets of mouse ESCs in this study, including a dataset for retinoid acid induced differentiation [15] and four datasets for spontaneous differentiation of four ESC lines (three lines from [22]; one unpublished, S.Z. and W.H.W, manuscript in preparation). We restricted the analysis to the regulatory relationships among 747 genes that are annotated by Gene Ontology term Transcription Regulator Activity, and are present on the Affymetrix U72av2 array. We designated six known TFs, Oct4, Sox2, Nanog, Klf4, Esrrb and Tcl1 as regulators of this system, due to their previously characterized role in ESCs. Interaction-Identifier [14] was applied to each time course microarray dataset. A list of common TF Interaction forms across datasets was then generated by Evidence merger. Genes were then grouped by their predicted regulators as well as their roles of regulation, i.e. activator and repressor. Twelve gene groups were formed. ChIP-chip data are available for Oct4, Sox2, Nanog and Klf4. Five out of eight regulatory-target relationships involving these four regulators were significantly enriched with ChIP-chip verified relationships (Table 1). RNA knock-out experiments were performed for all the six regulators [15,17]. Nine out of twelve target gene groups involving these six regulators were enriched with RNAi verified regulatory relationships (Table 2). Note that when using RNAi data for testing the predicted regulatory role of a TF, we only counted the target genes whose changes of expression were in the consistent direction to the predicted role of its TF, but not counting all targets genes with any changes to both directions. These tests demonstrated that the predicted regulatory relationships were in general consistent to those derived from independent experiments.

**Table 1:** Validation by ChIP-chip data

Role	TF	# of target genes	# of genes verified in ChIP	Chi-Square	P-value
Activation	Nanog	39	12	10.46986	**0.00121**
	Sox2	121	21	12.437	**0.00042**
	Oct4	67	8	2.436113	0.11857
	Klf4	49	18	13.90787	**0.00019**

Repression	Nanog	47	11	4.190152	**0.04066**
	Sox2	132	19	5.778738	**0.01622**
	Oct4	103	11	2.121288	0.145264
	Klf4	62	14	1.335151	0.247891

**Table 2:** Validation by RNA interference data

Role	TF	# of target genes	# of genes verified in RNAi	Chi-Square	P-value
Activation	Nanog	39	9	9.710604	**0.00183**
	Sox2	121	20	16.22083	**5.6E-05**
	Oct4	67	13	25.26604	**5E-07**
	Esrrb	95	6	2.966206	0.085021
	Tcl1	21	2	4.650429	**0.03105**
	Klf4	49	16	25.21262	**5.1E-07**

Repression	Nanog	47	2	0.018713	0.891192
	Sox2	132	12	9.035917	**0.00265**
	Oct4	103	7	3.909397	**0.04802**
	Esrrb	73	6	5.407721	**0.02005**
	Tcl1	27	2	4.663594	**0.03081**
	Klf4	62	10	0.537394	0.463515

**Figure 1:**
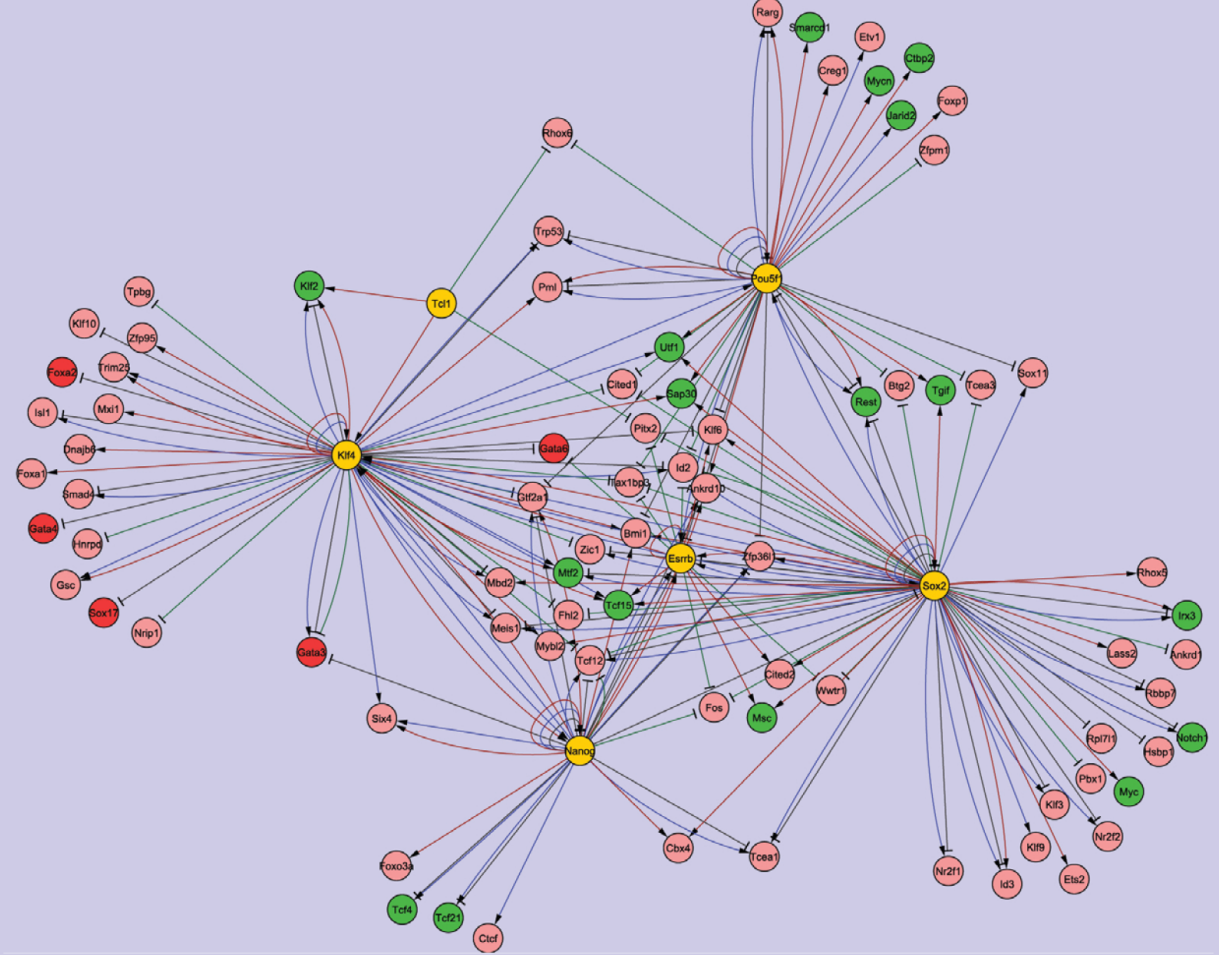
**The gene regulatory network identified by Network-Identifier**. Yellow nodes represent regulators. Green nodes represent genes promoting self-renewal and pluripotency. Red nodes represent genes used for differentiation. Sharp and blunt arrows represent activation and repression effects, respectively. Red and green lines represent activation and repression activities with RNAi evidence, respectively. Blue and black lines denote regulatory relationships with ChIP-chip evidence.

Finally, Network-Identifier identified the regulatory relationships that were predicted by expression data and had consistent evidence from either RNAi or ChIP-chip data. We used Cytoscape [23] to display the final reported regulatory relationships (Figure 1).

87 regulators and target genes were reported in the ESC transcription network (Figure 1). In particular, the mutual regulation of Klf2 and Klf4 were recently shown to be an important module for maintaining the undifferentiated state of ESCs [17]. Utf1 and Myc are known to be key ESC transcription factors. The result that they are under the control of Oct4 and Klf4 underscores the importance of Klf4 in promoting self-renewal. Mtf2 has only recently been implied to inhibit differentiation by recruiting the polycomb group of transcription repressors [24]. This analysis indicates that Klf4 and Sox2 could synergistically activate Mtf2 in ESCs. The regulatory relationships for a number of genes involved in lineage specific differentiation were also identified. These include Gata6, Gata3, Sox17 and FoxA2. Inhibiting these lineage specific differentiation genes in ESCs is critical to maintain an undifferentiated state. Among the predicted network, there were a number of transcription repressors, including Ctpb2 and Rest. Ctpb2 was predicted to be activated by Oct4. Rest was predicted to be jointly regulated by Oct4 and Sox2. These results suggest that Oct4 and Sox2 could indirectly inhibit differentiation genes by activating transcription repressors such as Ctpb2 and Rest.

## Discussion

Network-Identifier is proposed to reconstruct transcription network based on biophysical models of transcription regulation. Multiple temporal gene expression datasets are used as inputs to Network-Identifier. ChIP-chip and RNAi data can also be utilized by Network-Identifier as independent validation datasets to further improve the predicted networks. Moreover, Network-Identifier has great flexibility in incorporating independent datasets other than ChIP-chip or RNAi data to reinforce the strength of validation.

It should be recognized that there are still a number of simplifications made in the modeling of the biophysical properties of gene regulation. A number of molecular events are not included in the model. These include: 1) the interactions of more than two TFs, 2) long range interaction of enhancer binding TFs and RNAP, 3) DNA methylation and 4) chromatin structure and state. Future work that takes these molecular features and events into account will potentially improve the accuracy of network reconstruction.

## Methods

### Revisiting the Interaction-Identifier method

**Figure 2:**
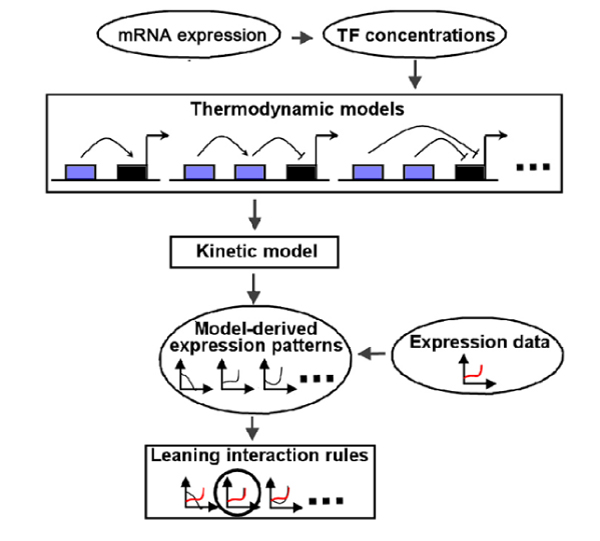
Flowchart of the Interaction-Identifier method.

We previously described the Interaction-Identifier method for identification of the candidate form of interaction among TFs and RNAP on the promoter of a target gene [14]. Interaction-Identifier models how a given TF interaction form affects the transcript concentrations of a target gene at steady states. Searching the space of TF interaction forms, it identifies the form that minimizes the difference between model-derived target concentrations and the observed expression data. The method is composed of three components: (1) a thermodynamic model for translating a TF interaction form and TF concentrations into the equilibrium probability of RNAP binding to the promoter of the target gene; (2) a kinetic model for derivation of steady state transcript concentrations of the target gene; and (3) matching gene expression data to model-derived steady state concentrations and identifying the underlining TF interaction form (Figure 2).

#### Thermodynamic models for RNAP binding

Thermodynamic models are based on the assumption that the level of gene expression is proportional to the equilibrium probability that RNAP binds to the promoter of interest; and these probabilities can be computed in a statistical mechanics framework. The Interaction-Identifier method follows recent efforts [12,25,26] to translate TF concentrations into the equilibrium probability of RNAP binding using thermodynamic models.

Considering the transcription of the target gene is regulated by only one TF on its promoter, a promoter can then take one of the four possible states: (1) both the TF and the RNAP bind with the promoter; (2) Only the RNAP binds to the promoter; (3) Only the TF binds to the promoter; (4) neither the TF nor the RNAP binds with the promoter, where let the weight of promoter with no RNAP or TF be 1 and the weights *q*_*p*_, *q*_*TF *_and *w*_*TFp*_*q*_*p*_*q*_*TF *_denote the ratios between the probabilities of states 2, 3, 4, respectively. The probability of the promoter of the target gene being bound with an RNAP is:

$$\text{RNAP is}:\frac{q_{p}+w_{TFp}q_{TF}q_{p}}{1+q_{p}+q_{TF}+w_{TFp}q_{TF}q_{p}},\text{where }w_{TFp}=\{\begin{array}{cc} 1 & \text{No interaction}\\ 10\sim 100 & \text{Activation}\\ 0 & \text{Repression} \end{array}$$

A TF can serve as either an activator or a repressor, or simply it does not interact with the RNAP, represented by different *w*_*TFp*_. These effects can be simulated by choosing appropriate *w*_*TFp*_. If *w *is set to 1, it represents that RNAP and the TF independently bind to the promoter. If *w *is set to 10~100, it represents that the TF helps to recruit RNAP to the promoter. The larger *w *is the higher the synergism is. If *w *is set to 0 or close to 0, it represents that the TF blocks the RNAP binding, and thus the TF is a repressor.

Under the statistical mechanics framework, similar expressions can be derived for genes with two regulatory TFs capable of binding to a promoter together with RNAP. By adjusting the interaction factors *w*, we can obtain an analytical form for the probability of RNAP binding under different forms of interactions among RNAP and the two TFs.

#### A kinetic model for equilibrium transcript concentration

The equilibrium concentration of the transcripts of a target gene is governed by its synthesis and degradation rates. Empirical data show that mRNA synthesis rate is proportional to the equilibrium probability of RNAP binding to promoters [12,25,26]. Interaction-Identifier further assumes that mRNA degradation rate is proportional to its concentration, which seems to be a reasonable assumption based on recent studies [27,28]. However the authors can always relax this assumption into more general forms at the model evaluation and model improvement stages. Empirical data on eukaryotic mRNA synthesis and degradation are available to estimate these rates [27-29]. Ordinary equations are used to implement this kinetic model.

### Inferring gene regulatory network

**Figure 3:**
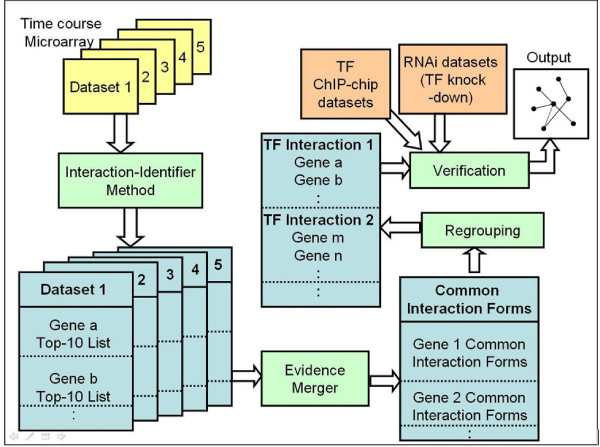
**Flowchart of inferring a gene regulatory network**. Input microarray datasets are shown in yellow and independent experimental data for validation are marked in orange. Intermediate results are shown in blue. Computational components are shown in green.

A computational framework for inferring gene regulatory networks (Network-Identifier) was developed based on thermodynamic modeling of transcription regulation. Network-Identifier requires more than one time course microarray experiments for the same biological process as input datasets. The method has three components: 1) Interaction-Identifier [14], 2) Evidence merger and 3) Verification component (Figure 3). For each time course dataset, Network-Identifier enumerates all possible regulatory forms on each target gene. These interaction forms include the activation or repression by a single TF, and the five interaction forms between any two TFs. Network-Identifier evaluates the fitness of each interaction form with Interaction-Identifier and ranks them according to their fitness. The 10 most likely interaction forms of TFs on a target gene are recorded as the Top-10 List. A built-in cutoff (default = 0.8) for Interaction-Identifier eliminates any interaction that is not well supported by data. It is therefore possible for a target gene to have less than 10 candidate TF interaction forms in its Top-10 List. The Top-10 Lists from every dataset are passed onto Evidence merger, which searches for the most frequently appeared interaction form in the Top-10 Lists of a target gene. This most frequently identified interaction form is passed onto the verification component. The verification component groups target genes according to their TF interaction forms. For each regulator-target relationship, for example TF-1 represses gene a, the target genes grouped into this relationship are subject to statistical tests. Chi-square tests are used to test whether the identified TF-target relationships are enriched with regulatory relationships identified from independent experimental data, such as ChIP-chip and RNAi data. Finally, if the tests are all insignificant, Network-Identifier will fail to report any regulatory network. If some of these tests are significant, suggesting there is consistency between the expression-derived regulatory relationships and those found by independent methods, Network-Identifier will invoke a compromise algorithm to report the regulatory relationships that are confirmed by at least two independent data sources. Currently the implemented compromise algorithm is to require the regulatory relationship identified by expression data to be reproduced in at least one of the two other experiments: ChIP-chip and RNAi. It is easy to substitute this algorithm with more sophisticated algorithms [30] or when some of the independent data are not available.

## Competing interests

The authors declare that they have no competing interests.

## Authors' contributions

CCC implemented the algorithm and performed data analysis. SZ initiated and supervised the project. CCC and SZ wrote the manuscript.
